# Comparison of continuous versus intermittent enteral feeding in critically ill patients: a systematic review and meta-analysis

**DOI:** 10.1186/s13054-022-04140-8

**Published:** 2022-10-25

**Authors:** Aaron J. Heffernan, C. Talekar, M. Henain, L. Purcell, M. Palmer, H. White

**Affiliations:** 1 Department of Intensive Care Medicine, Logan Hospital, MetroSouth Hospital and Health Service, Meadowbrook, QLD Australia; 2 School of Medicine and Dentistry, Griffith University, Southport, QLD Australia; 3 Royal Brisbane and Women’s Hospital, Brisbane, QLD Australia; 4 Faculty of Medicine, University of Queensland, Brisbane, QLD Australia

**Keywords:** Enteral nutrition, Intensive care unit, Gastric residuals

## Abstract

**Background:**

The enteral route is commonly utilised to support the nutritional requirements of critically ill patients. However, there is paucity of data guiding clinicians regarding the appropriate method of delivering the prescribed dose. Continuous enteral feeding is commonly used; however, a bolus or intermittent method of administration may provide several advantages such as minimising interruptions. The purpose of this meta-analysis is to compare a continuous versus an intermittent or bolus enteral nutrition administration method.

**Methods:**

A systematic review and meta-analysis were performed with studies identified from the PubMed, EMBASE, Cochrane Library and Web of Science databases. Studies were included if they compared a continuous with either an intermittent or bolus administration method of enteral nutrition in adult patients admitted to the intensive care unit. Study quality was assessed using the PEDro and Newcastle–Ottawa scoring systems. Review Manager was used for performing the random-effects meta-analysis on the outcomes of mortality, constipation, diarrhoea, increased gastric residuals, pneumonia, and bacterial colonisation.

**Results:**

A total of 5546 articles were identified, and 133 were included for full text review. Fourteen were included in the final analysis. There was an increased risk of constipation with patients receiving continuous enteral nutrition (relative risk 2.24, 95% confidence interval 1.01–4.97, *p* = 0.05). No difference was identified in other outcome measures. No appreciable bias was identified.

**Conclusion:**

The current meta-analysis has not identified any clinically relevant difference in most outcome measures relevant to the care of critically ill patients. However, there is a paucity of high-quality randomised controlled clinical trials to guide this decision. Therefore, clinicians may consider either dosing regimen in the context of the patient’s care requirements.

## Introduction

Nutritional support is an essential part of managing the critically ill patient. Critical illness is associated with catabolic stress, which increases the risk of multiorgan dysfunction, prolonged hospitalisation and increased morbidity and mortality [1, 2]. Early (< 48 h) progressive initiation of nutrition supplementation in critically ill patients with appropriate protein provision is likely to lead to reduced catabolism, improved gastrointestinal tract integrity and improved outcomes [3–6]. Enteral (EN) nutrition has several advantages when compared with total parental nutrition (TPN) and nutrition omission [7]. EN does not require central venous line access, thereby removing concerns of line compatibility and sufficient access. The aim of EN is to supply nutrients to improve immune system functioning [8–10]; preserve gastrointestinal integrity to prevent bacterial translocation [7, 8, 11] and optimise mucosal host defences; reduce muscle catabolism, and decrease mortality. Moreover, both TPN and EN are generally considered equivalent in patient-oriented outcomes such as mortality early in the patient’s treatment course [12, 13]. For prolonged administration, TPN may be associated with increased infection complications [14]. Despite the widespread use and familiarity of EN, the optimal dosing method remains controversial.

EN is usually delivered as a continuous rate in the intensive care unit (ICU) [9, 15]; an approach consistent with recent guidelines [14]. Continuous infusions may be associated with a lower provision of nutrition compared with intermittent boluses in situations where nutrition administration requires cessation to facilitate investigations or assess for extubation [14, 16]. Moreover, continuous administration may restrict patient mobility and alter gastrointestinal hormone secretion, which may lead to long-term metabolic complications such as hyperglycaemia and insulin resistance [16]. Other metabolic advantages of intermittent EN administration may also include enhanced protein synthesis and adherence to the usual circadian rhythm variability of hormones such as ghrelin and insulin that may lead to increased skeletal muscle autophagy [17, 18]. Therefore, intermittent EN administration is an attractive alternative; however, there are concerns that intermittent administration may lead to increased diarrhoea in critically ill patients and an increased risk of feeding intolerance, as well as a possible risk of aspiration in some studies [19, 20]. Taken together, both intermittent and continuous administration may both be preferred depending on the clinical circumstances, thus making the initial choice difficult for clinicians in view of potentially conflicting evidence.

The purpose of this systematic review and meta-analysis is to compare the effects of continuous versus intermittent/bolus feeding in critically ill patients.

## Methods

### Search strategy

A search of the literature was carried out within the PubMed, EMBASE, Web of Science and Cochrane Library electronic databases. The following search phrase was used: ((enteral*) OR (nasogastric*) OR (gastric*) OR (tube*) OR (forced*)) AND ((continu*) OR (bolus*) OR (intermittent)) AND ((nutrit*) OR (feed*) OR (diet*) OR (intoleran*) OR (glycemi*) OR (glycaemi*) OR (insulin*) OR (residu*) OR (calori*) OR (aspira*) OR (vomit*) OR (distens*) OR (diarrh*) OR (malnutri*)) AND ((critical*) OR (intensive*)).

The search was limited using filters as appropriate to include articles published with human participants where possible and articles published in English from 1946 and the 1^st^ of February 2022.

### Inclusion and exclusion criteria

**Fig. 1:**
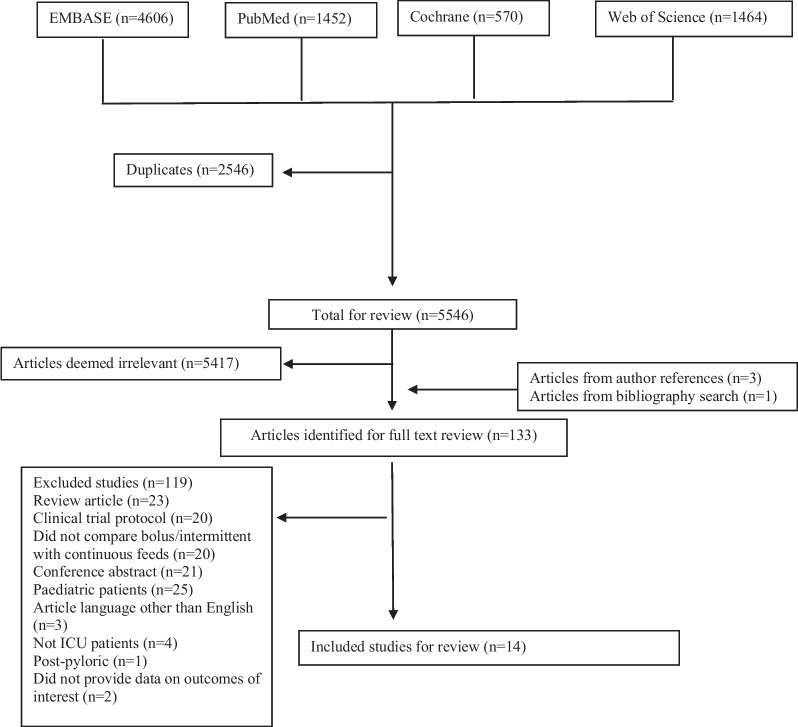
Flow diagram outlining article identification and selection

Published studies were included if they met the following inclusion criteria: (1) human participants admitted to an intensive care unit; (2) patients aged ≥ 18 y, and (3) the study compared an intermittent or bolus regimen with a continuous enteral feeding regimen using a pre-pyloric method (nasogastric or orogastric). Studies were excluded if: (1) the study was written in a language other than English; (2) involved animals, (3) included patients < 18 years of age, (4) was a conference abstract, (5) compared intermittent and bolus nutrition delivery methods, (6) the study included post-pyloric feeding methods, and (7) the manuscript was a case study or meta-analysis. Cohort, case–control, cross-over, and randomised controlled trials were included. Articles were selected for full text review based on the title and abstract. A summary of the review is presented in the Preferred Reporting Items for Systematic Reviews and Meta-Analyses (PRISMA) flow chart (Fig. 1).

### Data extraction

The following study details were extracted where possible from included studies: study year, participant demographic details, diagnosis/cohort group, study type, details of allocation concealment, blinding details where relevant, percentage of patients with full data for analysis, and details of the nutrition intervention. Outcome variables included mortality, diarrhoea, constipation, nosocomial pneumonia, increased gastric residuals and bacterial colonisation. Outcome variables were defined per the specific article. Article identification, evaluation, and data extraction were performed by two independent reviewers (MH and AH). Disagreements were decided by consensus with a third reviewer (HW and CT).

### Study methodological quality assessment and statistical analysis

Study methodological quality and bias was assessed using two scoring systems. RCTs were assessed using the PEDro scoring system and cohort studies assessed using the Newcastle–Ottawa Scoring system.

Meta-analysis was performed using Review Manager version 5.3. Data collected were number of patients with the outcome of interest. For dichotomous variables, the odds ratio and 95% confidence interval were determined. Heterogeneity was assessed using the *I*^2^ test. Only random effects models were utilised for analysis. Publication bias was assessed using Funnel Plots for each outcome. A *p* value < 0.05 was considered statistically significant.

## Results

### Study characteristics

A total of 8092 studies were identified with 2546 duplicates (Fig. 1). Of these 133 were included for full text review yielding 14 publications which met the inclusion criteria (Table 1). A total of 408 and 414 patients were included for the continuous, or intermittent/bolus regimens, respectively. Patients were typically admitted to a mixed or trauma/neurology ICU. Studies generally excluded patients with prior gastrointestinal complaints or with peritonitis. Only one study was not a randomised controlled study. Caloric estimates were generally based on a 25–30 kcal/kg/day requirement. Nutritional requirement outcomes were reported in only 4 studies, ranging from 23 to 82% of those included who met the prescribed intake and was consistent between groups. Similarly, illness severity scores were only reported in 7 studies, with average APACHE II scores ranging from 13 to 22.

**Table 1:** Included study details

References	Patient population	Continuous patients	Intermittent patients	Bolus patients	Continuous dose	Intermittent dose	Bolus dose	Caloric estimate	% Continuous target goal	% Intermittent target goal	% Bolus target goal	APACHE II continuous	APACHE II intermittent	APACHE II bolus	Study type	PEDro score	NOS
Kocan and Hickisch [21]	Neurology/neurosurgical ICU	17	17	0	< 120 mL/h	< 370 mL/h 1 h infusion Q4H	NA	Wilmore nomogram	62.2	55.9	NA	ND	ND	NA	RCT	4	NA
Bonten et al. [22]	Mechanically ventilated mixed ICU	30	30	0	< 83.3 mL/h	< 111.1 mL/h 18 h infusion	NA	ND	ND	ND	ND	19 (13–23)^a^	17 (13–22)^a^	NA	RCT	6	NA
Chen et al. [23]	Mechanically ventilated mixed ICU	51	0	56	Q24H	NA	20 min infusion Q4H to Q6H	25 kcal/kg/day	ND	ND	ND	13^a^	NA	14^a^	RCT	6	NA
Evans et al. [24]	Neurology/ Neurosurgical ICU	24	0	26	Q24H	NA	ND	ND	ND	NA	ND	14.6 (4.26)	NA	14.0 (4.37)	RCT	7	NA
Maurya et al. [25]	Neurology/ Neurosurgical ICU	20	20	NA	Q24H	NA	Unspecified infusion rate Q3H	30 kcal/kg/day	ND	NA	ND	ND	NA	ND	RCT	7	NA
Gowardman et al. [26]	Mixed ICU	12	15	0	Q24H	18 h infusion	NA	30 kcal/kg/day	53	23	NA	20	15	NA	RCT	8	NA
MacLeod et al. [27]	Trauma ICU	81	79	0	Q24H	30–60 min infusion Q4H	NA	25 kcal/kg/day	58.3	60.2	NA	14 (10–17)^a^	12 (9–16)^a^	NA	RCT	8	NA
McNelly et al. [28]	Mechanically ventilated mixed ICU	59	0	62	Q24H	NA	5 min infusion Q6H	Penn-State equation or 25 kcal/kg/day	72.5	NA	82.4	20.2 (18.2–22.3)^a^	NA	23.1 (19.9–26.2)^a^	RCT	9	NA
Nasiri et al. [29]	Mixed ICU	30	0	30	18 h/day	NA	20 min infusion Q3H	Harris-Benedict equation	ND	ND	ND	ND	ND	ND	RCT	6	NA
Shahriari et al. [30]	Mixed ICU	25	0	25	Q24H	NA	20 min infusion Q6H	Harris-Benedict equation	ND	ND	ND	ND	ND	ND	RCT	6	NA
Serpa et al. [31]	Mixed ICU	14	14	0	Q24H	1 h infusion Q3H	NA	25 kcal/kg/day	ND	ND	ND	ND	ND	ND	RCT	7	NA
Spilker et al. [32]	Mechanically ventilated mixed ICU	13	13	0	Q24H	18 h infusion	NA	ND	ND	ND	ND	ND	ND	ND	Case–Control	NA	6
Steevens et al. [33]	Mixed ICU	9	0	9	Q24H	NA	15 min infusion Q4H	25–30 kcal/kg/day	ND	ND	ND	ND	ND	ND	RCT	6	NA
de Araujo et al. [34]	Mixed ICU	23	18	0	Q24H	18 h infusion	NA	25–30 kcal/kg/day	ND	ND	ND	22.4 (6.05)	20.7 (4.95)	NA	RCT	7	NA

*ICU* intensive care unit, *NA* not applicable, *ND* not described, *NOS* Newcastle Ottawa Score, *Q24H* administered every 24 h, i.e. continuous, *Q3H* administered every 3 h, *Q6H* administered every 6 h, *Q4H* administered every 4 h, *RCT* randomised controlled trial

^a^Median and interquartile range

The risk of bias in included studies varied with most studies having a moderate risk of bias, predominantly due to an absence of blinding and allocation concealment.

### Outcomes

**Fig. 2:**
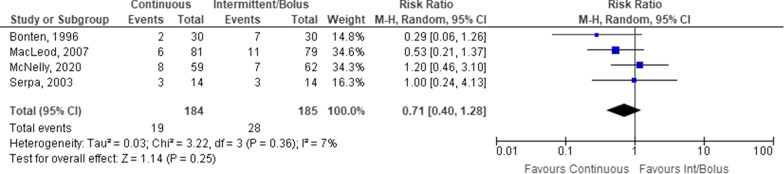
Mortality difference in patients receiving continuous versus intermittent/bolus enteral nutrition

Overall, there was only a difference between continuous and intermittent/bolus administration in constipation rates, with no difference in other outcomes. Mortality was described in four studies of a total of 369 study participants (Fig. 2). No statistically significant difference was identified between intermittent/bolus and continuous EN.

**Fig. 3:**
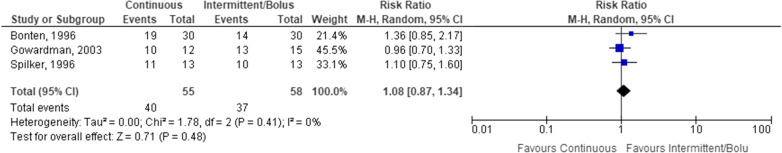
Patients colonised with potentially pathogenic bacteria receiving continuous versus intermittent/bolus enteral nutrition

There was no statistically significant difference in the number of patients colonised with potentially pathogenic bacteria in either the oropharynx or upper gastrointestinal tract, although only 3 studies of a total of 113 participants were included (Fig. 3).

**Fig. 4:**
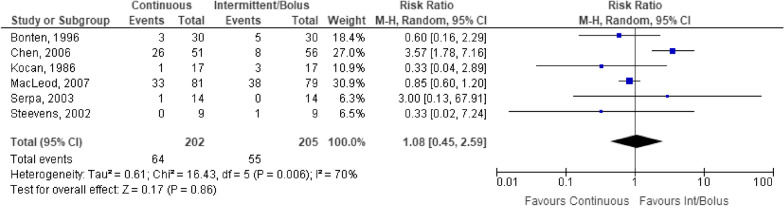
Patients developing pneumonia receiving continuous versus intermittent/bolus enteral nutrition

Six studies of 407 participants examined pneumonia as an outcome (Fig. 4). No statistically significant difference was identified between administration methods (Fig. 4). Sensitivity analysis by removing the Bonten et al. [22] study that defined an intermittent infusion as that administered over 18 h did not change the outcome (OR 1.25, 95% CI 0.31–5.08, *p* = 0.75). There was considerable heterogeneity in outcome that may be due to the variable definitions of pneumonia (Fig. 4). Pneumonia was variably defined, but the presence of blue dye in respiratory secretions was the most common method of detection.

**Fig. 5:**
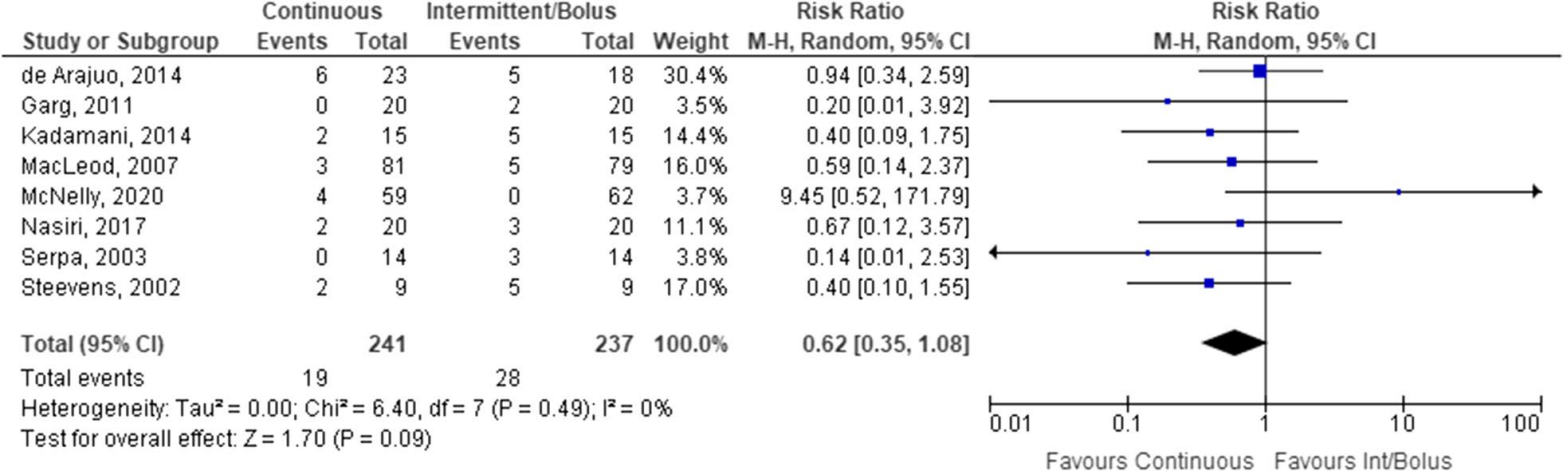
Patients with diarrhoea receiving continuous versus intermittent/bolus enteral nutrition

There was no statistically significant difference between administration methods for gastrointestinal disturbance including diarrhoea (Fig. 5), or increased gastric residuals (Fig. 7). Diarrhoea was assessed in 8 studies with a total of 478 study participants. No statistically significant difference was identified between continuous and intermittent/bolus EN routes. Removing the study conducted by de Araujo et al. [34] that defined intermittent administration as 18 h/day did not change the outcome significantly (OR 0.46, 95% CI 0.20–1.05).

**Fig. 6:**
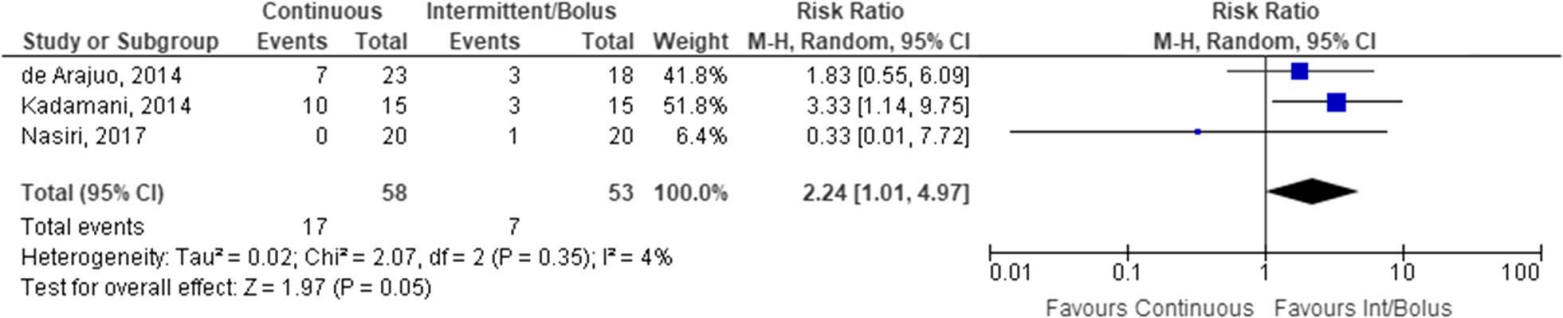
Patients with constipation receiving continuous versus intermittent/bolus enteral nutrition

In contrast to diarrhoea, constipation was only assessed in 3 studies consisting of 111 participants. There was a statistically significant difference between continuous and intermittent/bolus EN, with an increased relative risk of constipation in patients receiving continuous EN (relative risk = 2.24, 95% CI 1.01–4.97, *p* = 0.05) (Fig. 6).

**Fig. 7:**
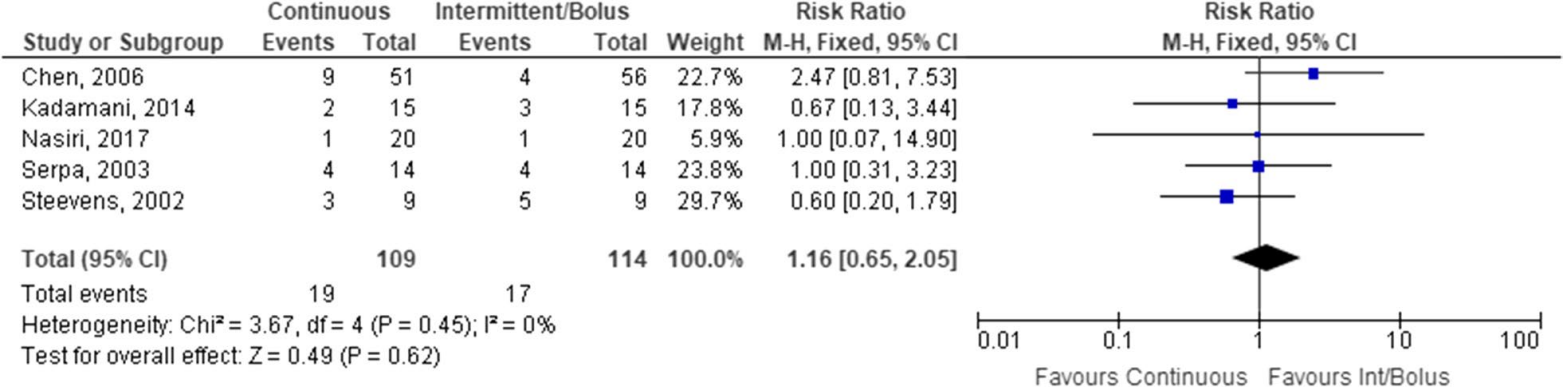
Patients with increased gastric residuals receiving continuous versus intermittent/bolus enteral nutrition

Gastric residuals were assessed as an outcome in 5 studies (*n* = 223). No statistically significant difference was observed between intermittent/bolus and continuous EN (Fig. 7). Gastric residual volumes > 150–300 mL assessed every 3–4 h were considered excessive across included studies.

Other outcomes such as glycaemic variability were assessed in two studies, but did not have standardised outcomes precluding meta-analysis. McNelly et al. assessed the incidence of hypo- and hyperglycaemia. No patients in either arm became hypoglycaemic. In contrast, 50% and 33.3% of patients in the intermittent and continuous arms became hyperglycaemic (blood glucose concentration > 10.1 mmol/L), respectively. Shahriari et al. compared the average blood glucose concentration between groups, finding no statistically significant difference (131.31 vs. 140.26 mg/dL for continuous and intermittent EN groups, respectively). Three studies compared gastric pH. Overall, there was no appreciable difference between intermittent/bolus and continuous EN administration (Table 2).

**Table 2:** Gastric pH

References	Gastric pH continuous	Gastric pH intermittent
Bonten et al. [22]	2.2 (IQR 1.3–3.9)	3.5 (IQR 1.8–5.2)
Gowardman et al. [26]	5	4
Spilker et al. [32]	4.7 (SD 0.5)	3.8 (SD 0.6)

### Bias assessment

**Fig. 8:**
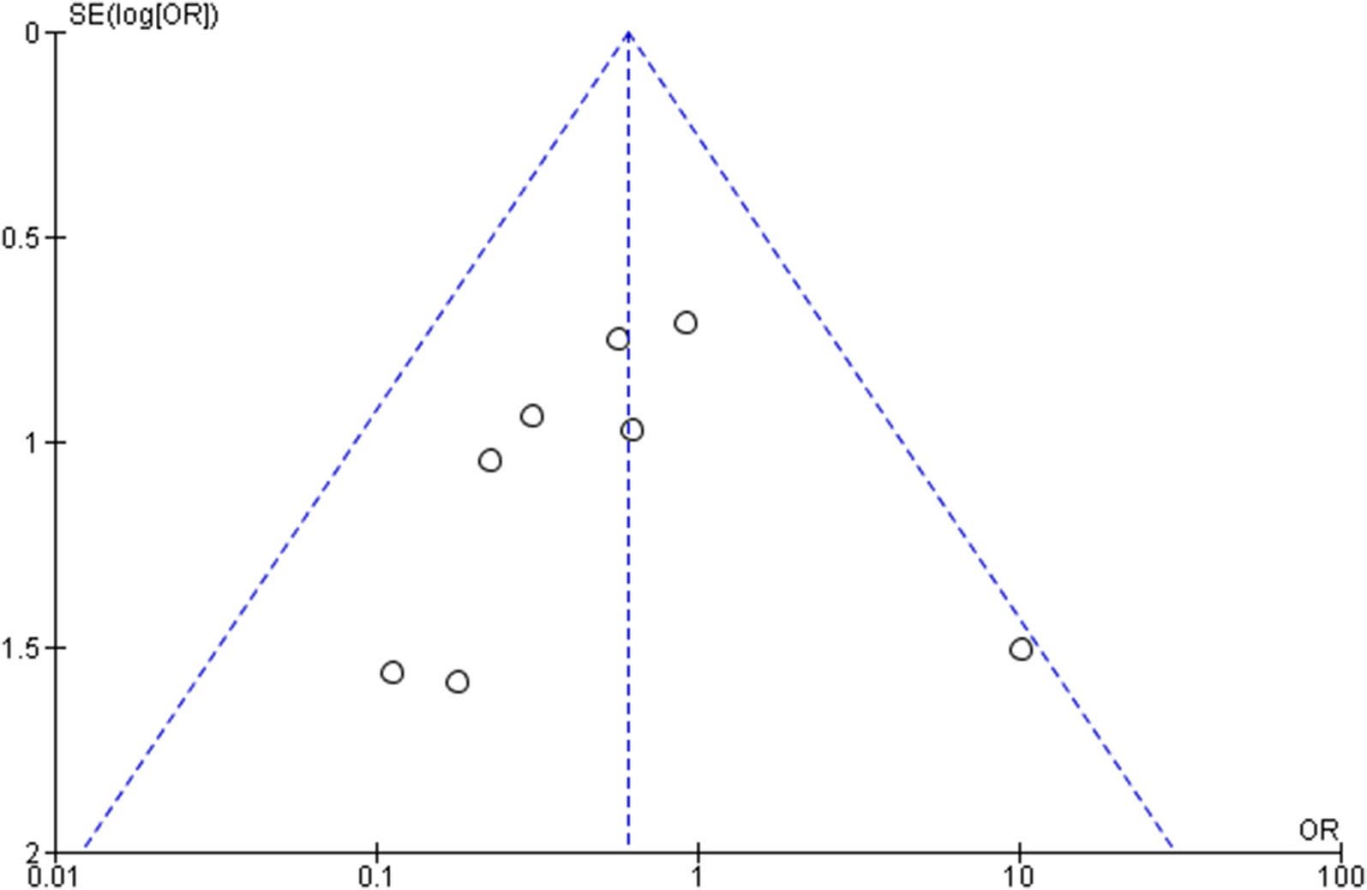
Funnel plot for diarrhoea outcome assessment

There was no appreciable bias as assessed by funnel plots. The Funnel plot assessing diarrhoea is depicted in Fig. 8 as a representative sample.

## Discussion

The aim of this review was to compare continuous versus intermittent feeding in critically ill patients. Outcomes assessed included bacterial colonisation, gastrointestinal disturbance (diarrhoea or constipation), increased gastric residuals, pneumonia incidence and mortality. Overall, our meta-analysis identified that there was an increased risk of constipation in patients receiving continuous infusions of EN. There was no statistically significant difference in any other outcome.

In clinical practice, it is widely accepted that both continuous feeding and bolus/intermittent feeding are practical and effective methods of administering the prescribed diet [26]. Current evidence suggests that each administration method may have its own adverse event profile and given the lack of long-term outcome data, clinicians may therefore select the method to mitigate such adverse events [17]. A recent meta-analysis of four studies (*n* = 236) identified that continuous administration reduced the risk of diarrhoea when compared with bolus EN administration (Risk Ratio 0.42, 95% CI 0.19–0.91), but did not identify any difference in other outcomes [14]. The subsequent guideline recommendation was therefore to recommend continuous EN administration on this basis, which contrasts with our own findings showing no difference in diarrhoea between administration methods. The current study included the same studies, but also included additional studies that met the inclusion criteria. A separate meta-analysis by Ma et al. [19] did not support these findings but found that continuous administration was associated with constipation, which is similar with that of our own findings. Our analysis was otherwise consistent with the findings of Ma et al. [19]. Indeed, other pragmatic issues may alter the administration method of the prescribed diet. Intermittent/bolus administration may be associated with increased daily caloric delivery by an average of 184.81 kcal compared with continuous administration [19]. This was not observed in our study but is conceivable due to the increased likelihood of continuously administered EN being interrupted for patient cares and diagnostic testing [35]. Additionally, bolus/intermittent administration is more likely to achieve nutritional goals in a shorter time frame, given the commonly used practice of slowly initiating continuous EN [24]. The impact of this on patient outcomes remains unclear. The use of intermittent or bolus administration may also allow the patient to mobilise without additional tubing minimising mobility in the hospital environment.

Bolus administration may also have additional metabolic advantages, although studies are limited. Animal models suggest that optimal protein synthesis occurs within 90 min of feeding, with approximately double the protein synthesis observed in neonatal pigs administered intermittent boluses compared with a continuous infusion [36, 37]. Similarly, in healthy human studies, an amino acid bolus stimulated increased protein synthesis compared with a continuous infusion [38]. Modulating the administration of EN may be advantageous to optimise protein administration given that a negative protein balance and reduced protein supplementation have been associated with increased functional disability and mortality in high risk critically ill patients [39–41]. Additionally, other hormones may be adversely affected by continuous EN administration. Glucose-dependent insulinotropic polypeptide (GIP) and glucagon-like peptide-1 (GLP-1) are both decreased in response to continuous EN administration, which may lead to increased insulin resistance, increased hepatic steatosis and may, in part, explain increased muscle catabolism in critically ill patients who receive continuous EN [42, 43]. However, current clinical evidence would suggest that neither method of feeding affects a patient’s resting energy expenditure and short term blood sugar concentrations, but data are limited [25]. Both feeding methods may give rise to some form of gastroenterological short-term complications in critically ill adults. Therefore, current practice should balance these potential adverse events for individualised patient care to mitigate potential adverse events.

Our study is not without limitations. First, only a small number of moderately biased studies with limited patient numbers are available that preclude the conclusions that may be drawn. Second, there was a paucity of studies examining the impact of continuous or bolus EN administration on the short-term and long-term metabolic impact. Third, only articles written in English were reviewed. Fourth, the outcome definitions assessed varied between studies, likely reflecting the variability in current practice. Fifth, study definitions for increased gastric residual volumes are conservative (> 300 mL) relative to contemporary practice (> 500 mL), which may influence the interpretation of the results. Sixth, there is a lack of data presented by the study authors detailing the determination of the patient’s nutritional requirements, formulas used, and additional influences, such as the inclusion of propofol in nutrition calculations. Last, there were variable definitions of intermittent, bolus and continuous feeds as presented in included studies. Our study defined these terms in accordance with the study definitions.

## Conclusion

This review compared the two methods of EN (continuous feeding and intermittent feeding) in critically ill patients. Unfortunately, there is a paucity of data for the Intensive care clinicians to determine which feeding method is best for their patients. Further research is needed to evaluate which feeding method achieves better nutritional goals and recovery, metabolic function and has least short-term complications.

## Data Availability

The datasets used and/or analysed during the current study are available from the corresponding author on reasonable request.
